# Normalization of alcohol misuse and alcohol-related harms: a mixed methods analysis exploring alcohol misuse, morbidity, and healthcare engagement in people experiencing homelessness

**DOI:** 10.1093/alcalc/agaf071

**Published:** 2025-12-01

**Authors:** Catherine Wells, Rachel Dewar-Haggart, Kate Glyn-Owen, Hannah Stevens, Julie Parkes, Yun Kim, Ryan M Buchanan

**Affiliations:** Primary Care, Population Sciences and Medical Education, Faculty of Medicine, Tremona Road, University of Southampton, Southampton, SO16 6YD, United Kingdom; Public Health and Social Research Unit, West Sussex County Council, West Street, Chichester, PO19 1RG, United Kingdom; Primary Care, Population Sciences and Medical Education, Faculty of Medicine, Tremona Road, University of Southampton, Southampton, SO16 6YD, United Kingdom; Nuffield Department of Primary Care Health Sciences, University of Oxford, Woodstock Road, Oxford, OX2 6GG, United Kingdom; Primary Care, Population Sciences and Medical Education, Faculty of Medicine, Tremona Road, University of Southampton, Southampton, SO16 6YD, United Kingdom; University Hospital Southampton, Tremona Road, Southampton, SO16 6YD, United Kingdom; Primary Care, Population Sciences and Medical Education, Faculty of Medicine, Tremona Road, University of Southampton, Southampton, SO16 6YD, United Kingdom; Primary Care, Population Sciences and Medical Education, Faculty of Medicine, Tremona Road, University of Southampton, Southampton, SO16 6YD, United Kingdom; Primary Care, Population Sciences and Medical Education, Faculty of Medicine, Tremona Road, University of Southampton, Southampton, SO16 6YD, United Kingdom

**Keywords:** homelessness, alcohol, alcohol-related liver disease, healthcare

## Abstract

**Aim:**

To understand the views and experiences of homeless adults who drink hazardously around alcohol use, alcohol harms and access to liver healthcare, and to quantify the prevalence of alcohol-related morbidity in this population.

**Methods:**

A sample of homeless adults (aged 18+) who drink hazardously (AUDIT score ≥8) were recruited to complete a health and alcohol use survey. From this sample, a smaller sample was purposively selected for semi-structured interview. Participants were recruited via liver outreach clinics held in five homeless hostels/day-centres in Southampton. Using a critical realist approach, qualitative data were analyzed using reflexive thematic analysis and descriptive statistics produced for survey responses.

**Results:**

Around 56 survey participants were recruited, 84% of whom had probable alcohol dependence and 18% a diagnosis of advanced liver fibrosis/cirrhosis. Themes identified from 10 interviews described the ubiquity of alcohol misuse and harms in the life-histories of people experiencing homelessness (PEH), the differing levels of understanding and risk recognition of alcohol-related harms, and how PEH rationalize hazardous drinking, despite the risks. Normalization of alcohol misuse and harms underlies these themes and likely contributes to feelings of fatalism and powerlessness to prevent these harms.

**Conclusions:**

Normalization of alcohol-related harms may represent a barrier to timely engagement with healthcare and a mechanism driving greater likelihood of alcohol-related harms in PEH. Improving knowledge around alcohol-related harms and healthcare may help to counter the misperceptions of risk and fatalistic attitudes that normalization fosters. Such intervention may be particularly effective for PEH if targeted towards those accessing hostels and day-centres.

## Teaser

**Short Summary:** Interviews with homeless adults who drink harmfully or dependently showed alcohol misuse and harms to be ubiquitous in their social environments and thus normalized. Normalization of alcohol-related harms may represent a barrier to timely engagement with healthcare and a mechanism driving greater likelihood of alcohol-related harms in homeless adults.

## Introduction

Alcohol misuse is a major driver of ill-health, disability and premature mortality in the UK (Williams *et al*. 2020). At particular risk of alcohol harms are people experiencing homelessness (PEH), a group which includes those who sleep rough, sofa-surf or live in hostels or temporary accommodation (European Federation of National Organizations Working with the Homeless 2017).

PEH experience significant health inequalities, including greater morbidity (Bowen *et al*. 2019, Lewer *et al*. 2019) and premature mortality rates estimated to be more than double those of the general population (Fazel *et al*. 2014, Aldridge *et al*. 2018). In the UK, the average age of death of PEH is an estimated 43–45 years (Office for National Statistics 2022) and nearly a third of deaths are attributable to causes preventable with more timely healthcare (Aldridge *et al*. 2019).

PEH are more likely to experience alcohol dependence compared to the general population (Fazel *et al*. 2014), with one small study estimating 50% prevalence of alcohol dependence in PEH accommodated in hostels in England (Hashim *et al*. 2022). The disproportionate health impacts of alcohol misuse are evident in the high burden of alcohol-related hospital admissions, morbidity and mortality reported for PEH in the UK (Field *et al*. 2019, Hashim *et al*. 2022, Office for National Statistics 2022). Studies identify alcohol as the main contributor to a fifth of emergency hospital admissions (Field *et al*. 2019), whilst the prevalence of liver disease – a condition most commonly caused by alcohol use (Karlsen *et al*. 2022) – is estimated at 26% (Hashim *et al*. 2022). As liver disease is often asymptomatic and diagnosed at a late or fatal stage (Karlsen *et al*. 2022), this is likely an underestimate. An estimated 9.6% of homeless deaths in the UK are due to alcohol-specific causes (Office for National Statistics 2022), predominantly due to alcohol-related liver disease (ARLD) (Office for National Statistics 2021). Deaths where alcohol is a contributing factor, such as cancers made more likely by alcohol or liver disease, further inflate the burden of alcohol-related harms amongst PEH.

Premature deaths from ARLD are preventable with early identification and intervention (Karlsen *et al*. 2022). However, amongst PEH, timely engagement with health services is poor: PEH are significantly less likely to be registered with a general practitioner than the general population (Elwell-Sutton *et al*. 2017) and instead access emergency healthcare at 60-times the rate of the general population (Bowen *et al*. 2019). Emergency re-admissions are also more frequent in PEH (Lewer *et al*. 2021). PEH report numerous barriers in accessing healthcare (McNeill *et al*. 2022), which can contribute to a perceived lack of control over health (Mc Conalogue *et al*. 2021) and lead to feelings of hopelessness and fatalism (O'Carroll and Wainwright 2019).

Despite the high burden of ill-health and mortality associated with alcohol use amongst PEH, the perceptions of PEH around their alcohol-related health needs, behaviours and beliefs is under-researched. How these perceptions intersect with ARLD prevalence and access to healthcare is also unquantified.

Using a mixed methods approach, this study aims to understand the prevalence of alcohol-related morbidity and the views and experiences of alcohol use and healthcare in homeless adults who consume alcohol at a hazardous level.

By exploring drivers for alcohol misuse, the burden of alcohol-related morbidity and barriers to care, this study hopes to inform the development of healthcare services for this vulnerable population.

## Methods

Using a critical realist approach, this study used an embedded mixed methods design (Creswell and Plano Clark 2017), with a qualitative study embedded in a larger quantitative study. To ensure transparency, this study was guided by the Standards for Reporting Qualitative Research checklist (O'Brien *et al*. 2014).

### Inclusion criteria and sampling

A sample of homeless adults (aged ≥18 years) with hazardous, harmful or dependent alcohol consumption (AUDIT score ≥8), who were able to communicate and provide informed consent in English were recruited via Liver Outreach Clinics hosted in five sites across Southampton, England: four homeless hostels and one homeless day-centre. Initial access to these participants was via hostel/day-centre managers, who referred residents. To mitigate potential selection bias associated with manager referral, recruitment was supplemented by participant peer-referral to the study. It was hoped this could encourage more marginalized and less engaged people to participate.

Participants were recruited to complete a closed-question, interview-based survey about their health, alcohol use and social networks. From within this sample, participants were purposively selected for semi-structured interview, using a heterogenous sampling strategy to ensure a balance of interviewees across sex, age, liver disease status and recruitment site.

**Figure 1:**
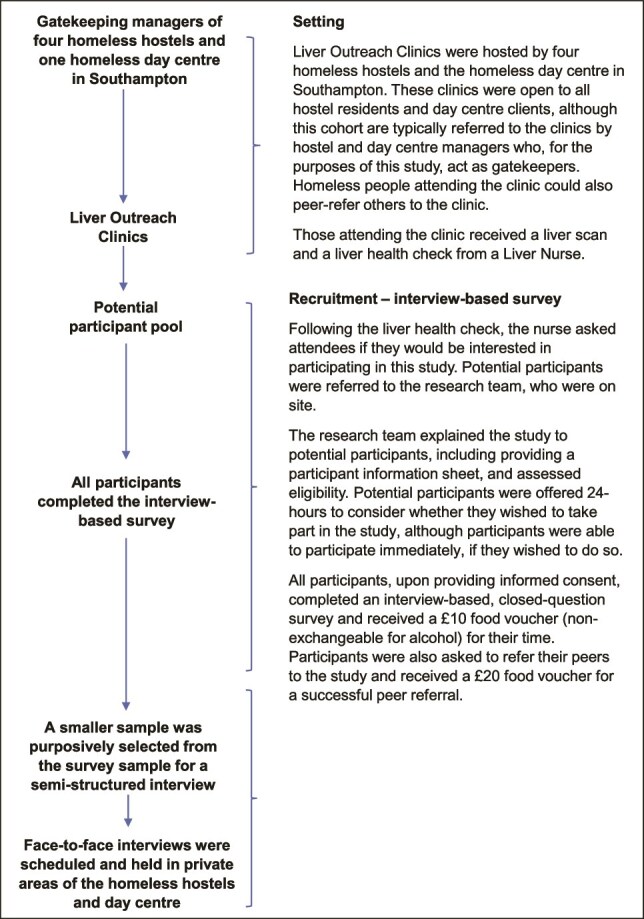
Recruitment strategy

For each part of the study, potential participants were provided with a participant information sheet and written consent was required. Participants received a £10 food voucher for survey completion and another for completing an interview. Participants who successfully referred one peer to the study received an additional £20 voucher (Fig. 1).

### Data collection

All participants completed the survey (Supplementary Material), which was developed with a patient and public involvement and engagement (PPIE) representative group at one face-to-face meeting and piloted with three attendees at the homeless day-centre to ensure face validity. The survey incorporated validated measures for alcohol consumption [AUDIT (Babor *et al*. 2001)] and comorbidity [Charlson co-morbidity index (CCI) (Charlson *et al*. 1987)].

The semi-structured interviews were face-to-face, held in private areas of the recruitment sites and led by one researcher (RB) using a topic guide (Supplementary Material). Interviews were audio-recorded, transcribed verbatim and anonymized.

Participants were recruited and data collected throughout February–July 2022.

### Data analysis

Survey data were summarized using descriptive statistics in SPSS v29.0.2.0. Interview transcripts were analyzed by one researcher (CW), supported by peer debriefing with two others (RB and RDH). To support discussion of CW’s initial ideas and interpretation, RDH independently coded two transcripts. Transcripts were triangulated against survey data describing participants’ alcohol intake, demographics and health. Reflexive thematic analysis (Braun and Clarke 2006) was used to enable an exploratory and interpretative analysis of rich but discursive interviews. An abductive approach to coding and searching for themes was used (Pope and Mays 2020), enabling inductive generation of themes grounded in the data, whilst acknowledging the influence of previous research and theories in shaping the study aim and researcher’s perspective. Analysis proceeded iteratively, with continual refinement of codes and themes. Data were organized using NVivo v14.

Ethical approval was granted by the NHS Research Ethics Committee (North West – Preston Research Ethics Committee REC ref. 20/NW.0325) and the University of Southampton Faculty of Medicine Ethics Committee (ERGO reference: 94151).

## Results

A total of 56 participants completed the survey and 10 participants completed semi-structured interviews, the latter lasting 17–43 min (mean = 27 min).

Of the survey sample, 84% had probable alcohol dependence and 18% evidence of advanced liver fibrosis/cirrhosis (Table 1). The age-adjusted CCI was low, at 1.5 (SD 1.53), but multimorbidity and risk factors for non-alcohol related morbidity were common. Forty percent had a diagnosis of chronic obstructive pulmonary disease (COPD) or asthma, 91% currently smoked tobacco and 71% had a diagnosed psychiatric co-morbidity (anxiety, depression, or schizophrenia) (Table 2). Table 1 provides the balance of participant characteristics, between the subset who completed a semi-structured interview and the overall survey sample.

**Table 1:** Characteristics of semi-structured interview and survey participants.

**Characteristic**	**Interview Participants (n = 10)**	**%**	**Overall characteristics (including interview and survey participants) (n = 56)**	%
**Sex**				
Female	3	30	9	16.1
Male	7	70	47	83.9
**Mean age years (SD)**	52		44	
	(SD 5.85)		(SD 10)	
**Ethnicity**				
White British or Irish^a^	10	100	50	89.3
White Gypsy or Irish Traveller	0	0	2	3.6
Any other White Background	0	0	2	3.6
Any other Mixed / Multiple ethnic background	0	0	1	1.8
Black Caribbean	0	0	1	1.8
**AUDIT score** ^**b**^				
Hazardous or harmful drinking (8–19)	2	20	9	16.1
Probable dependence (≥20)	8	80	47	83.9
**Advanced liver fibrosis/cirrhosis** ^***^	3	30	18	32.1
**Recruitment site**				
Hostel A – ‘wet house’	3	30	8	14.3
Hostel B – ‘wet house’	1	10	10	17.9
Hostel C	1	10	6	10.7
Hostel D	2	20	22	39.3
Day centre	3	30	10	17.9

^*^^*^^*^Defined as a liver stiffness >12 kPa *measured using a Fibroscan™*.

^a^White British includes White English, Welsh, Scottish, Northern Irish, British or Irish*.*

^b^AUDIT – Alcohol Use Disorder Identification Test. An AUDIT score of 8 or above was a study inclusion criterion.

**Table 2:** Self-reported clinical service engagement and comorbidities in the interview based survey participants.

**Clinical engagement or comorbidities**	**n/56**	**%**
Current tobacco smoker	51	(91.1)
COPD	7	(12.5)
Asthma	15	(26.8)
Psychiatric diagnosis (e.g. depression or schizophrenia)	40	(71.4)
History of deliberate self-harm	29	(51.8)
Attempted suicide	27	(48.2)
Previous injecting drug use	5	(8.9)
Previous withdrawal seizures	27	(48.2)
T2DM	2	(3.6)
Registered with GP	56	(100)
Homeless healthcare GP	25	(44.6)
Current alcohol support	16	(28.6)
Hospital detox	21	(37.5)
Community detox	19	(33.9)
Emergency department attendance last 6 months [median (IQR, range)]	0	(0–1, 0–12)

IQR, interquartile range; T2DM, type 2 diabetes mellitus; detox, medically supported detoxification from alcohol; COPD, chronic obstructive pulmonary disease; GP, general practitioner.

Thematic analysis identified four themes and 12 sub-themes (Table 3). Themes are presented with illustrative quotations, with participant pseudonyms and, for context, liver health status (including self-reported diagnoses), and supported with survey results.

**Table 3:** Themes and subthemes from participant interviews.

**Theme**	**Sub-theme**
**Pathways into, and normalization of, alcohol misuse**	‘There was always alcohol in the house’ – Formative experiences
	‘Everyone was drinking’ – Ubiquity, normalization, and social contagion of drinking
	‘They just want to forget things’ – Coping and getting by
**Understanding and recognizing the risks of alcohol harms**	‘I did hear about that it affects your liver’ – Awareness of alcohol health harms
	‘It’s just one of them things’ – Normalization of alcohol harms
	‘I don’t think I drink a lot, but I know I do’ – recognizing risks to oneself
**Rationalizing drinking, despite the risks**	‘I’m going to live it in a way that I want to live it’ – Inaction after liver health screening
	‘It’s a catch-22’ – Powerlessness and lack of control over health
	‘It was down to me whether I drank’ – Control and choice in drinking
	‘Proper alcoholics’ – Comparing ‘levels’ of drinking
**Supportive and caring professionals**	‘If I was left on my own, I’d drink myself to death’ – Bridging barriers and gaps in capacity
	‘They listen to you’ – Care and respect

### Pathways into, and normalization of, alcohol misuse

#### ‘There was always alcohol in the house’ – Formative experiences

Most participants described alcohol as a prominent feature of their childhoods. Parental alcohol misuse or dependence was experienced as a norm by many, often against a backdrop of other adverse childhood experiences. Several participants described early initiation of drinking which, for some, quickly developed into harmful alcohol use.

‘I've been drinking since the age of 13. My mum and dad drunk. There was always alcohol in the house […] it was abusive […] so I left home when I was 15.’ Paul, liver fibrosis

#### ‘Everyone was drinking’ – Ubiquity, normalization, and social contagion of drinking

In adulthood, heavy or frequent alcohol use was a constant and normalized presence throughout participants’ living, working and socializing environments. Most participants were surrounded by a social circle of drinkers, and for some, the social pressure to partake in the group drinking behaviour appeared to act like a social contagion. Where participants were receiving constant encouragement from their peers, passive and active, to sustain their drinking, some expressed helplessness in managing their alcohol intake.

‘I mean it's really difficult to stop drinking when half your family drink, when everyone around you drinks […]. It's just like I can't get away from it.’ Tracy, normal liver

Some participants’ social networks appeared to exclusively comprise drinkers who, when money was tight, actively facilitated each other’s drinking. For these participants, alcohol appeared to be the glue in their social network, but these social bonds were ultimately fragile. This was evident where the removal of alcohol – either by community members giving up drinking or dying – resulted in these networks growing smaller over time.

*‘*People moved on and went separate ways. Some people I've known have ended up dying […] Some people come off the drink and you lose contact that way.’ Mark, liver cirrhosis

Findings from the wider survey sample support this theme. Participants reported a mean of 5.71 (SD 5.89) friends or family members who drink every day or almost every day, most of whom drink the same or more than them (i.e. also drinking hazardously or more). A total of 57% stated that they usually drink with others and, when purchasing alcohol, 38% stated that they buy alcohol to share with others.

#### ‘They just want to forget things’ – Coping and getting by

Participants often described difficult and traumatic experiences, including bereavements, becoming homeless and ill-health, for which alcohol was used as a coping tool. Alcohol was also used to pass time; however, implicit in this desire to drink the time away were deeper difficulties or dissatisfaction in life for which alcohol was again used to cope. This was alluded to where participants referred to the ‘job’ of alcohol, for which, as tolerance built up, more alcohol was needed to attain the same numbing result.

*‘*Why do people smoke, drink and take drugs? They just want to forget things, don't they?’ Alistair, liver fibrosis

In the survey sample, life difficulties were apparent in the high proportion of participants with histories of prison-time (75%), self-harm (52%), and suicide attempts (48%), alongside psychiatric co-morbidities (71%).

### Understanding and recognizing the risks of alcohol harms

#### ‘I did hear about that it affects your liver’ – Awareness of alcohol health harms

Alcohol-related harms were prevalent in the survey sample. Eighteen percent were known to have advanced liver fibrosis/cirrhosis, 48% had experienced alcohol withdrawal seizures and 38% hospital detoxification. On average, participants had 1.6 (SD .5) close friends or family with previous ARLD-related hospital admissions.

All interview participants showed awareness of the health risks of excess alcohol consumption, including ARLD. This knowledge often seemed to stem from personal or peers’ experience, including several participants who referred to their own ARLD.

‘I had a brain haemorrhage because the alcohol weakened the vessels in my head […] I've seen my brother die from alcohol, his pancreas bursting and that, and a couple of my friends’. Scott, liver cirrhosis

Many participants volunteered their knowledge of liver damage when discussing their relationship with alcohol and health. In others, particularly those with little personal or peer experience of ARLD, understanding of alcohol health harms was more uncertain, indicating low health literacy around ARLD.

*‘*I did hear about that it affects your liver […] I don't really know about the liver, I just know that people end up in hospital and can die from it.’ Christine, normal liver

#### ‘It’s just one of them things’ – Normalization of alcohol harms

Throughout the interviews, there was a casualness to participants’ descriptions of what were objectively traumatic and life-changing experiences of alcohol-related illnesses, accidents and deaths. The frequency with which participants had experienced or heard about alcohol-related harms seemed to have normalized these harms.

‘When I was living in [that area] there was a big lot of us drinking, a lot of them are dead now […] either from being intoxicated, got hit by motors, got run over. It's just one of them things.’ John, liver fibrosis/cirrhosis

This normalization similarly applied to the social harms of alcohol misuse, including blasé attitudes towards imprisonment and difficult family relationships. In several instances, these social harms manifested in participants losing the conventional support network of their close family, likely replaced by the more fragile social networks of drinkers described previously.

#### ‘I don’t think I drink a lot, but I know I do’ – Recognizing risks to oneself

Most participants recognized that their past or current drinking was excessive, and many had recently reduced their alcohol intake. However, despite an awareness of alcohol-related health risks, many participants appeared not to recognize this risk of harm in *themselves*. This was apparent as a perceived invulnerability, and even invincibility, to alcohol harms in some. In others, low recognition of alcohol-related risks to their health seemed related to low health literacy around alcohol harms.

*‘*Honestly no, [I don’t worry how drinking has affected my health] because I am not drinking to be drunk.’ Jack, normal liver

Other participants *did* seem to recognize the personal health risks of alcohol misuse but were avoiding facing the possible – or actual – consequences of their drinking. This was particularly apparent in those with ARLD, whose responses appeared to minimize and disbelieve the severity of their diagnosis.

*‘*There's no problem with [my liver] at this moment, but if I carry on drinking obviously it will become a problem.’ Alistair, liver fibrosis

### Rationalizing drinking, despite the risks

#### ‘I’m going to live it in a way that I want to live it’ – Inaction after liver health screening

When asked about their experience of the liver health scan offered during study recruitment, all participants showed appreciation for the benefits of liver screening, including early identification of issues and easing anxieties around symptoms. However, when asked how the scan or other liver health investigations had affected them, few participants indicated any effect on their alcohol intake. Two participants felt unable to change their behaviour, whilst another reflected on the cost–benefit balance of quitting drinking.

‘I question myself is it worthwhile giving up drinking to try and save my liver? If I get told that I've only got a certain amount of time to live then I'm going to live it in a way that I want to live it […] I wouldn't give up alcohol just because there's a chance to save my life.’ Alistair, liver fibrosis

#### ‘It’s a catch-22’ – Powerlessness and lack of control over health

Where participants already had an ARLD diagnosis, many perceived limits to their self-efficacy to protect their health. Some described a lack of control over their health, due to the ‘*catch-22*’ between ARLD and alcohol withdrawal seizures, or seemed to justify their ongoing drinking, despite ARLD diagnoses, with fatalism. In some, a belief in chance or a higher power seemed to account for this powerlessness to change their behaviour and health state.

‘I'm only allowed one bottle a day. If I don't have that, I'll get rattles and have fits. So I have that and slowly die or don't have it and die quicker. Sod's law, isn't it?’ Scott, liver cirrhosis

#### ‘It was down to me whether I drank’ – Control and choice in drinking

Feeling a lack of control was apparent in several participants’ descriptions of struggling to manage their alcohol intake. Where participants did indicate some control, this related to choosing to *start* drinking or to accept an offered drink. Self-reflection on past behaviours, including acknowledgement of personal responsibility for drinking decisions, was evident in some participants’ accounts, particularly in those with an ARLD diagnosis.

*‘As my mum would've said, you can lead a horse to water but you can't make it drink; they used to offer me it and it was down to me whether I drank, wasn't it*’ Sally, liver cirrhosis

#### *‘*Proper alcoholics’ – Comparing ‘levels’ of drinking

When discussing their ongoing drinking, several participants conceptualized their alcohol intake in terms of different ‘levels’ of drinking, with the implication that some levels were more acceptable than others. Participants seemed to use this ‘levels’ concept as a framework within which they could rationalize their ongoing drinking – often despite ARLD diagnoses. In doing so, comparisons were made to their own past drinking and to their peers’ drinking, who were perceived to be at greater risk of harm than themselves.

‘I'm keeping my drinking to what I see as a respectable level from what I used to drink.’ Alistair, liver fibrosis

‘Yes, I've seen people, proper alcoholics, where they've got so bad that they were drinking thinners and stuff. I don't do anything like that.’ Sally, liver cirrhosis

These comparisons highlighted the distorted reference point that participants were comparing themselves against, within their social reality where excess drinking was the norm.

### Supportive and caring professionals

Of the survey sample, 32% were living in a ‘wet house’ hostel where they received a daily measure of alcohol. Around 45% were registered with a specialist homeless primary care service and 30% were currently engaged with community alcohol support.

#### ‘If I was left on my own, I’d drink myself to death’ – Bridging barriers and gaps in capacity

Support professionals in community settings (CSPs) held an important role in helping many participants to manage their drinking, including monitoring of daily alcohol intake in hostel settings. Several participants seemed to welcome this external control of their alcohol intake, whereby hostel staff acted to bridge the gap across some participants’ seemingly underdeveloped capacity for self-control.

‘They manage it here for me. I get four pints a day which stops me from rattling. It's a good place to be. If I was left on my own, I'd drink myself to death.’ Scott, liver cirrhosis

CSPs also supported participants to manage their healthcare needs, by bridging barriers to access, such as poor memory resulting in missed appointments, and encouraging engagement with health screening.

#### ‘They listen to you’ – Care and respect

In encounters with healthcare and community professionals, several participants had felt stigmatized and disrespected because of their alcohol dependence. Consequently, participants appeared to value caring and respectful attitudes from these professionals, without judgement for their histories or current situations.

‘[In another area, it seemed] that they didn't care about you because you are an alcoholic, whereas down here it's nothing like that. They sit, they listen to you.’ Sally, liver cirrhosis

Being treated as valued individuals, worthy of understanding and concern, also appeared to facilitate participants’ self-management of their drinking, including instances where CSPs had supported participants to engage in meaningful activities and thus control their drinking.

## Discussion

### Summary

This study of homeless adults who drink alcohol hazardously explored experiences of alcohol use, alcohol-related harms, engagement with care, and the prevalence of alcohol-related morbidity. Themes describe the ubiquity of alcohol misuse and alcohol-related harms in the life-histories of PEH, the differing levels of understanding and risk recognition of alcohol-related harms, and how PEH rationalize hazardous drinking, despite the risks.

Underlying these themes is the normalization of alcohol misuse and harms amongst PEH. Normalization starts in childhood with parental alcohol misuse and continues in the social drinking environments and networks of adulthood, which perpetuate excess drinking as the norm. Alcohol-related accidents, illnesses and deaths are similarly normalized by their prevalence in the communities of PEH and likely contribute to feelings of fatalism and powerlessness to prevent these harms.

### Normalization of health-harming behaviours and adverse consequences in social environments

Systematic reviews show that parental drinking behaviour is associated with children’s drinking (Rossow *et al*. 2016) and that adults view their parents’ drinking as instrumental in shaping their own drinking behaviours and attitudes (Muhlack *et al*. 2018). Normalization of these harmful behaviours for the children involved is implicit. Considering the common accounts of parental alcohol misuse and other adverse childhood experiences (ACEs) in PEH provided here and elsewhere (Mabhala *et al*. 2017, Mc Conalogue *et al*. 2021), and the far higher prevalence of ACEs amongst PEH compared to the general population (Liu *et al*. 2021), this population is likely at greater risk of harmful behaviours being normalized in the social environments of childhood.

The apparent social contagion of drinking and communities of homeless drinkers described here are also reflected in studies showing that alcohol consumption behaviours cluster in social networks, including homeless communities (Wenzel *et al*. 2009). With evidence for selective choice of social connections who also drink and peer influence in drinking behaviours (Rosenquist *et al*. 2010), social networks may therefore contribute to entrenching harmful behaviours, particularly amongst socially excluded homeless communities.

Although few studies explore the perceptions of PEH around alcohol use and harms, comparable evidence exists for the normalization of other health-harming behaviours and their consequences in PEH. Smoking, for example, is described as a community activity amongst PEH, perpetuated by peer pressure and proximity within social groups (Soar *et al*. 2020). The adverse consequences of harmful behaviours are also normalized in other marginalized populations, such as the fatalistic attitudes of street-based sex workers (SBSW) towards violence from clients (Gorry *et al*. 2010, Leaker and Dunk-West 2011) and of people who inject drugs (PWID) towards hepatitis-C infection (Wozniak *et al*. 2007, Skeer *et al*. 2018). In drug-injecting communities, the high prevalence of hepatitis-C normalizes this infection as an inevitable part of the injecting ‘lifestyle’, which PWID have no control in preventing (Wozniak *et al*. 2007, Harris 2009, Skeer *et al*. 2018). This echoes the normalization of alcohol-related morbidity within drinking communities and feelings of powerlessness over health identified here.

Although PWID and SBSW are not directly comparable to PEH, these groups share common experiences as marginalized populations: PWID and SBSW often experience homelessness and face social exclusion and disadvantage (Luchenski *et al*. 2018). Marginalization itself may be a driving factor in the normalization of harmful behaviours and their adverse consequences in these groups. The common experiences of stigma and exclusion may reinforce the selection of social networks with similar experiences (McPherson *et al*. 2001), thereby concentrating harmful behaviours and their consequences within these communities and perpetuating them as a norm.

### Normalization of harms as a barrier to services

**Figure 2:**
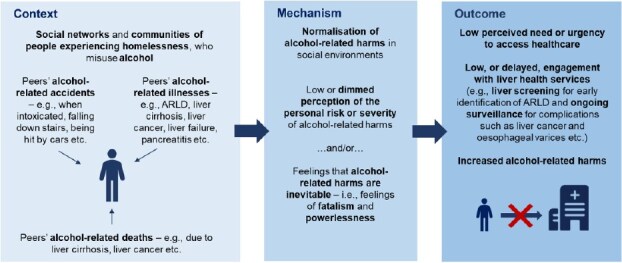
Normalization of alcohol-related harms as the mechanism by which hazardously drinking people experiencing homelessness have low engagement with liver health services and increased likelihood of alcohol-related harms, presented within the realist Context-Mechanism-Outcome framework

Using a critical realist lens and the associated ‘Context-Mechanism-Outcome’ (CMO) framework (Pawson and Tilley 1997), normalization of alcohol-related harms, as identified here, can be suggested as a mechanism by which hazardously drinking PEH have low engagement with liver healthcare and increased likelihood of alcohol-related harms. Because alcohol-related harms are normalized in their social worlds, PEH may have a dimmed perception of the risk or severity of these harms or may feel that alcohol-related harms are inevitable. This may result in low perceived need or urgency to engage with healthcare, including screening for early ARLD identification or ongoing surveillance. Normalization of alcohol-related harms may therefore represent a barrier to PEH’s engagement with liver healthcare and ultimately increase the risk of alcohol-related harms (Fig. 2).

This CMO suggestion is supported by systematic reviews identifying low perceived and normalized risk as barriers to hepatitis-C screening and treatment in PWID (Jones *et al*. 2014, Paisi *et al*. 2022). The relative importance of these factors is uncertain, however, when compared to other reported barriers to healthcare, including low health literacy, perceived stigma, and poor relationships with professionals – also identified here in hazardously drinking PEH.

### Strengths and limitations

This study benefits from a mixed methods approach to explore alcohol use and harms in PEH, with recruitment across multiple sites and qualitative findings triangulated against quantitative data in a larger sample. Given the small sample, data saturation was not achieved in the semi-structured interviews. However, as the analysis provided rich and novel insights from a marginalized population on an under-researched topic, this sample holds ‘information power’ as an exploration of experiences (Malterud *et al*. 2015).

Limitations include restricted eligibility, excluding PEH unable to communicate in English or provide informed consent, or who did not engage with the liver outreach clinic (i.e. the door to recruitment). As these characteristics may themselves represent barriers to accessing services, important perspectives may have been missed. There was also potential for response bias, as participants knew that the interviewer is a hepatologist, which may have prompted a perceived uneven power dynamic (Hinton and Ryan 2020) and encouraged answers perceived to be more ‘favourable’.

## Conclusions and implications

As a small mixed methods study, situated in the UK, conclusions about the experiences of all hazardously drinking PEH cannot be drawn. However, as an exploration of the experiences of some of society’s most marginalized voices, findings may be extended to other marginalized groups, who often share similar experiences of social exclusion and inequality (Luchenski *et al*. 2018).

Interventions to reduce alcohol misuse and harms in PEH may benefit from addressing harmful norms and expectations and by utilizing social networks. Education to address misperceptions around alcohol harms and healthcare may help to counter the low perceptions of risk and fatalistic attitudes identified here, which may consequently support timely engagement with services. For PEH, such approaches may be particularly effective if delivered via peers within social networks, as demonstrated in peer education and advocate programmes to improve uptake of hepatitis-C testing in PEH (Paisi *et al*. 2022) and to encourage harm reduction practices in drug-using communities (Li *et al*. 2012). Such interventions may be best targeted towards hostel/day-centre settings, where communities of homeless drinkers are clustered and thus relatively accessible.

Ultimately, the most impactful approach to combat alcohol-related harms lies in prevention. For many interview participants, parental alcohol misuse was normalized during childhood. In the UK, an estimated 472 000 children have an alcohol- or drug-dependent parent (Children’s Commissioner for England 2019), which is linked to numerous poor health and social outcomes (Hedges and Kenny 2018). Previous UK government-funded interventions targeted towards children of alcohol-dependent parents have improved identification and outcomes of this group (Department of Health and Social Care 2023). To reduce the risk of future health inequalities and social exclusion for this vulnerable population – including the risk of future homelessness and alcohol misuse – continuing and widening provision of such early interventions is essential.

## Supplementary Material

Supplementary_IBS_agaf071

Supplementary_materials_wells_agaf071

Supplementary_Topic_guide_1_agaf071

## Data Availability

Data available from authors on reasonable request subject to the ethical constraints of this study.
