# Supplementary material for: Normalization of alcohol misuse and alcohol-related harms: a mixed methods analysis exploring alcohol misuse, morbidity, and healthcare engagement in people experiencing homelessness
Source: Alcohol Alcohol. 2025 Dec 1;61(1):agaf071. doi: 10.1093/alcalc/agaf071 (PMC12667265; doi:10.1093/alcalc/agaf071)
Supplement: Supplementary_IBS_agaf071 [file supplementary_ibs_agaf071.docx]

Date…………………… Supervised by……………

Interview based survey

Study number:

**CIRCLE THE CORRECT ANSWER**

Thank you for agreeing to complete this survey. Your responses will be kept confidential and questions can be skipped on request.

PART 1: Background information

**1.3i What sex are you?**

1. Male
2. Female

**1.3ii How would you describe your gender?……………………….**

**1.4 How old are you?**

**1.5 What is your level of education?**

1. GCSE/O-levels
2. A-Levels
3. University
4. Apprentice
5. Left school before 16
6. Other…………………………………

**1.6 What is your status?**

1. Single
2. Married
3. Co-habiting
4. Civil partnership
5. Divorced

**1.7 Do you currently work?**

1. Full time
2. Part time
3. Temporary/variable work
4. Unemployed/sickness/disability benefits
5. Retired

**1.8 What is your ethnic group?
Choose one option that best describes your ethnic group or background**

**White**

1. English / Welsh / Scottish / Northern Irish / British 2. Irish
3. Gypsy or Irish Traveller
4. Any other White background, *please describe*

**Mixed / Multiple ethnic groups**

5. White and Black Caribbean
6. White and Black African
7. White and Asian
8. Any other Mixed / Multiple ethnic background, *please describe*

**Asian / Asian British**

9. Indian
10. Pakistani
11. Bangladeshi
12. Chinese
13. Any other Asian background, *please describe*

**Black / African / Caribbean / Black British**

14. African
15. Caribbean
16. Any other Black / African / Caribbean background, *please describe*

**Other ethnic group**

17. Arab
18. Any other ethnic group, *please describe*

**1.9 Have you spent time in prison?**

1. Yes
2. No

PART 2: Personal health

**2.0 Are you registered with a GP?**

1. Yes
2. No
3. Don’t know

If answer No or don’t know go to 2.3

**2.1 Is the GP based in Southampton?**

1. Yes
2. No

**2.2 If yes - Is it the Southampton Homeless Healthcare Team?**

1. Yes

2. No

**2.3 How many times have you been to AE in the last month?**

**2.4 How many times have you been to AE in the last 6 months?**

**2.5 The last time you went to AE what was it for?**

1. Accident/Trauma/Injury
2. Deliberate or accidental (non-alcohol) poisoning?
3. Alcohol excess/detox
4. 1&3
5. Liver disease
6. 3&5
7. Other
8. Not applicable

Other………………………………………………

**2.6 Please indicate which of the following apply to you (tick the box):**

| 1. Current smoker of tobacco |  |
| --- | --- |
| 1. Current smoker of cannabis/crack |  |
| 1. A diagnosed smoking related lung disease |  |
| 1. Asthma |  |
| 1. Active injecting drug use |  |
| 1. A diagnosed psychiatric illness e.g. depression, anxiety schizophrenia |  |
| 1. Previous self-harm without the intention of taking your own life |  |
| 1. A previous attempt to take your life e.g. overdose, attempted hanging |  |
| 1. Heart disease e.g. angina or a previous heart attack |  |
| 1. Active Tuberculosis |  |
| 1. Active Hepatitis B |  |
| 1. HIV |  |
| 1. Active cancer (undergoing treatment or active monitoring) |  |
| 1. Epilepsy |  |
| 1. Alcohol withdrawal seizures |  |
| 1. Diabetes mellitus |  |
| 1. Other   *Please describe:* |  |

**Part 3 – Alcohol & liver disease**

**2.6 Are you currently attending alcohol support services?**

1. Yes
2. No

If yes where?…………………………………………………

**2.7 Have you ever been detoxified for alcohol in hospital?**

1. Yes

2. No

**2.8 Have you ever been detoxified for alcohol in the community?**

1. Yes
2. No

**2.9 Have you ever been told you have liver cirrhosis (scarring of the liver)?**

1. Yes

2. No

**2.10 If yes – Have you attended for a (ring all that apply):**

1. Clinic appointment at the hospital?

2. Ultrasound scan at the hospital?

3. Endoscopy test (into the stomach) at the hospital?

4. None of the above

5. Not applicable

**2.11 Have you had a test for liver cirrhosis called a Fibroscan?**

1. Yes

2. No

**2.12 Have you had a test for Hepatitis C?**

1. Yes

2. No

3. Not sure

**2.13 Have you been told that you have Hepatitis C?**

1. Yes

2. No

3. Not sure

**2.14 – If yes - have you been successfully treated or are you currently seeing specialists about treatment?**

1. Yes

2. No

3. Not applicable

**2.15 Have you had a vaccination for Hepatitis B?**

1. Yes

2. No

3. Not sure

Part 3: Alcohol drinking behaviours

**3.0 When you last consumed alcohol were you?**

1. Alone

2. With one other person

3. With more than one other person

**3.1 Do you usually consume alcohol?**

1. Alone

2. With one other person

3. With more than one other person

**3.2 The last time you purchased alcohol was it?**

1. Just for you
2. For you and others

**3.3 When you usually buy alcohol is it?**

1. Just for you
2. For you and others

**3.4 When you last drank alcohol?**

1. Had you purchased/acquired it?
2. Had someone else purchased/acquired it?

**3.5 Do you have a close friend who drinks alcohol every day and starts drinking alcohol in the morning or who drinks the same amount as you?**

1. Yes
2. No

**3.6 Do you have a family member who drinks alcohol every day and starts drinking alcohol in the morning or who drinks the same amount as you?**

1. Yes

2. No

**3.7 Do you live or sleep close to someone who drinks alcohol every day and starts drinking alcohol in the morning or who drinks the same amount as you?**

1. Yes

2. No

**3.8 Do you have a close friend/family member who has been admitted to hospital because of liver disease in the past month?**

1. Yes

2. No

**3.9 If you buy alcohol in Southampton list the three shops where you purchase this (Name/Street e.g. Budgens/Highstreet):**

**1………………………………….**

**2…………………………………..**

**3………………………………… N/A**

**3.10 List the three drinks you buy most often:**

**1………………………………….**

**2…………………………………..**

**3………………………………… N/A**
