# Supplementary material for: Normalization of alcohol misuse and alcohol-related harms: a mixed methods analysis exploring alcohol misuse, morbidity, and healthcare engagement in people experiencing homelessness
Source: Alcohol Alcohol. 2025 Dec 1;61(1):agaf071. doi: 10.1093/alcalc/agaf071 (PMC12667265; doi:10.1093/alcalc/agaf071)
Supplement: Supplementary_materials_wells_agaf071 [file supplementary_materials_wells_agaf071.docx]

Supplementary materials

## **Positionality statements (authors leading the data collection, data analysis and drafting of the final manuscript)**

Of the study authors, CW and RB were primarily involved in the design, data collection and data analysis of this study. As part of this study’s critical realist and reflexive approach, and to support transparency, a reflection on the characteristics and relevant experiences of each researcher is provided below.

CW is a female, early career public health researcher from a middle-class background. CW has a professional background in local authority public health research, with a recent focus on homelessness, although she does not have professional experience of direct interaction with this population. During her MSc in Public Health, CW received training in qualitative research and applied these skills to qualitatively analyse the data explored in this study for her dissertation.

RB is a male Consultant Hepatologist and Clinical Academic Researcher, with experience in mixed methods research in marginalised populations. As a hepatologist, RB has expertise and a research interest in alcohol-related liver disease and in improving liver health outcomes in people from marginalised communities.

## Additional quotations

| **Theme** | **Sub-theme** | **Additional quotes** |
| --- | --- | --- |
| **Pathways into, and normalisation of, alcohol misuse** | “*There was always alcohol in the house*” – Formative experiences | *My dad was an alcoholic and my brother was an alcoholic. My mum used to have a drink, but she didn't drink every day […] they used to fight and what have you, and he used to hit my mum and that.* Sally, liver cirrhosis  *I'm an alcoholic, and I've been an alcoholic since I was 12 years old*. John, liver fibrosis/cirrhosis  *I've been drinking from a young age. I first went into detox when I was 19 for alcohol.* David, normal liver |
|  | “*Everyone was drinking*” – Ubiquity, normalisation and social contagion of drinking | *I've been doing building for ten years, eight years, and the thing is you're your own boss […] you're in different houses every single day, or different places, and there's shops around the corner and you think, I'll just go to the shop, get something to eat, grab a can. Before you know it, you've had one can, you've had two cans, you've had three cans, and everyone else is doing it as well.* Paul, liver fibrosis  *Hostel life, homeless life, any environment where people were drinking […]* *I suppose we were just sitting there in groups, and everyone was drinking, so you'd tend to sit out nice days and tend to not realise that you'd drunk more than what you intended*. Mark, liver cirrhosis  *Street drinkers, the ones that we mix with. We've all got our own little crew that we mix and drink with. It comes with the territory. If you're a drinker, you're a drinker, that's the way it's been*. John, liver fibrosis/cirrhosis  *Everywhere I've been, they've been drinkers everywhere, even the places I've lived, because you say you keep yourself-to-yourself, but it's quite difficult in a house keeping yourself-to-yourself when you've got lots of people coming and going. It's quite difficult not bumping into people, isn't it? Lock the door and don't come out!* Tracy, normal liver |
|  | “*They just want to forget things*” – Coping and getting by | *Detox at the age of 19 and then seven years I left there teetotal clean and then settled down and then [my partner] passed away and then me drinking again.* David, normal liver  *At that time I liked it, I suppose; it took up my time. I like drinking, I still have a drink here but you are monitored, whereas up there you're not monitored; you just drink for the sake of drinking*. Sally, liver cirrhosis |
| **Understanding and recognising the risks of alcohol harms** | “*I did hear about that it affects your liver*” – Awareness of alcohol health harms | *I've got, obviously, liver damage. I suffer with my belly and then slowing down a lot. I've ended up with bad feet because of it, so that generally slows me down a little bit.* Mark, liver cirrhosis  *When taken to the hospital, I still got the hallucinations. They told me that this is because of the alcohol, because I quit too fast*. Jack, normal liver  *I did hear about that it affects your liver. I didn't really pay attention to it about how much I drank.*  [Interviewer: What had you heard?]  *It goes off, doesn't it?*  [Interviewer: It goes off?]  *I don't know. I don't really know about the liver, I just know that people end up in hospital and can die from it.* Christine, normal liver |
|  | “*It's just one of them things*” – Normalisation of alcohol harms | *My mate had problems with a bit of internal bleeding, but he was quite old and seriously drunk. He'd down a whole bottle of vodka in one go and couldn't stop the bleeding. Another mate had a tumour which they couldn't operate on. That got the better of him eventually. Mate has lost his legs. A couple of mates lost their legs through drinking and so they've given up*. Mark, liver cirrhosis  *I heard the other day, one of the people I used to drink with, he got so drunk he fell down the stairs, knocked his head and killed himself.* Sally, liver cirrhosis  *I've been in trouble with the police, all that, but it comes with the territory. You have too much to drink and you think you're superman, you think you can take on the world, but it's just the alcohol*. John, liver fibrosis/cirrhosis  *I've not always gone out and ended up in the cells. I have actually come back and been okay. Unfortunately, the last time I did that my mother called the police on me, and that's why I ended up in prison […] Trusty good old mothers!* *Parents, hey, so that's why I wouldn't go back and live there anyway.* Tracy, normal liver |
|  | “*I don't think I drink a lot, but I know I do*” – Recognising risks to oneself | *I used to drink – well, I shouldn't be proud of it really – but I used to drink 16 cans of Special Brew. It's 9.8 per cent alcohol, which is quite strong. The most I drunk of that was 38 cans one day and then I passed out. Terrible, isn't it?* Scott, liver cirrhosis  [Interviewer: Going back to that [hospital admission], was that the only time you've ever been admitted, do you think, because of the excesses of drinking?]  *I'm 50 years old, I'm a very, very healthy man. Never broke a bone, except for my nose, never go to hospital. Never get flu […] I've got a friend […] he drank a lot, he's got emphysema and all that kind of shit now. He's bad. He's just not built like me*. Paul, liver fibrosis  [Interviewer: Do you know people that have been unwell [from liver disease]? […] What do you know?]  *They've gone yellow. They've ended up in hospital. They cough up blood and all sorts.*  [Interviewer: That's people you've known from around here? Did that have an impact on you?]  *No, because I don't understand it. I don't think I drink a lot, but I know I do. Over the years it's got worse.* Christine, normal liver  [Interviewer: if you had the [liver] scan and it showed there was some scarring on your liver, how would that affect you?]  *I don't know really. Until I'd had the test, I'd be like all right. So, what do you do? If you've got scarring of the liver, what, do you just stop drinking? People still don't do that, do they? It might frighten them a bit. I don't know. I've never really thought about it.* Tracy, normal liver  [Interviewer: If you were going to get some blood tests done or see a GP, what would that experience be like for you?]  *It'd be quite traumatic because if they were like, your liver's nearly had it, because I did have a scan with you, didn't I? I know it's not too clever, is it? I don't think, is it? That's life.*  [Interviewer: We can go through that with you at the end if you'd like.]  *No, it'll be all right […] What you don't know you don't think about, do you?* Scott, liver cirrhosis  *[My friends] did mention that I need to calm down off the cider and change it, but we do generally like lads' chat and all of that, a bit of friendly banter with it and stuff like that*. Mark, liver cirrhosis |
| **Rationalising drinking, despite the risks** | “*I'm going to live it in a way that I want to live it*” – Inaction after liver health screening | *So liver scan was good though because you can never tell what your liver is really like and that unless you get it checked over. Not just that. You could be healthy one minute and then the next minute you might not be healthy. So it's always good to get checked over*. David, normal liver  [Interviewer: How did both of you going through the scan change – did it change anything?]  *[My partner] has tried to cut down. He finds it hard, he shakes when he hasn't got a drink. I don't. I think he has cut down a bit since.* Christine, normal liver |
|  | “*It's a catch-22*” – Powerlessness and lack of control over health | *I don't want to end up having operations and I know I've got scarring so it's just trying to keep it down and keep it steady rather than being too mad and dying too early*. Mark, liver cirrhosis  *I'm always going to have that drink anyway so I'm not kidding myself that I'm not going to*. Alistair, liver fibrosis  *There's no way I pack up drinking. I still get seizures […] I've been told if I don't drink then you're going to get problems, so I have to drink, because your body needs it. It's not because you want it, it's your body telling you you need it, so it's a bit – it's a catch-22.* John, liver fibrosis/cirrhosis  *But you know, alcohol is a part of my life. I don't think I could ever… It's either stop drinking and have an alcoholic seizure or keep drinking and my liver bursts and my pancreas, so it's the lesser of two evils*. Scott, liver cirrhosis  *I'm fit as a fiddle, I just don't understand it. God hates me and wants me to live till about 300. I just never get ill. There's got to be a point, it's not like I've been kind to my body*. Paul, liver fibrosis  *When he wants you up there, your number's up, your number's up, isn't it?* Scott, liver cirrhosis  [Interviewer: Since having the scan, has that affected you in any way?]  *No, not really. I still drink. The damage is done, you can't repair it. It's one of them things, it's done, you've just got to carry on with it.* John, liver fibrosis/cirrhosis |
|  | “*It was down to me whether I drank*” – Control and choice in drinking | *Once I've had a drink I find it hard to put it down*. David, normal liver  *Nobody's come up and said, 'Right, let's go start drinking alcohol', it just don't happen. So it's always been my decision if I want to go and have something to drink, you go and have it to drink*. Alistair, liver fibrosis  *It's just one of them things you've got to deal with it yourself. There's no one who's put that can in your hand, it's you who's done it, so you've just got to get on with it*. John, liver fibrosis/cirrhosis |
|  | “*Proper alcoholics*” – Comparing “levels” of drinking | *I think I've gone too far now. As long as I keep it on a level and don't go like I used to.* John, liver fibrosis/cirrhosis  *My mate had problems with a bit of internal bleeding, but he was quite old and seriously drunk.* Mark, liver cirrhosis |
| **Supportive and caring professionals** | “*If I was left on my own, I'd drink myself to death*” – Bridging barriers and gaps in capacity | *You can drink here, but you're on a limit. You've got times when you can have a drink, times when you can't. My last drink's at eight o'clock at night, that's my last drink, and you don't get your fresh drink until ten o'clock, so you're monitoring how much you're drinking*. John, liver fibrosis/cirrhosis  *We used to sit there and drink, whereas here you don't. You are allowed a drink – obviously – but you only get so much drink; you're only allowed so much and they space it out, which is a lot better*. Sally, liver cirrhosis  *I've not missed any appointments down here. I've done the dentist, the doctors and everything, the hospital. I don't miss any appointments now because they write it all down in the office. They remind me the day before and they remind me again in the morning!* Sally, liver cirrhosis  *Staff told us to come down because they know that we drink, to go and get checked out [at the liver outreach clinic]. Otherwise I wouldn't have*. Christine, normal liver |
|  | “*They listen to you*” – Care and respect | *To be honest, it was quite a horrible experience because I wanted to go for a cigarette and the nurses were rude. Obviously, they knew I'd been in there for a drinking problem, they probably didn't have the respect*. Paul, liver fibrosis  *In the way that they approach you, in the way that they… Well, they listen to you, listen to what you've got to say, they're not making any comments about it.* Alistair, liver fibrosis  *She always comes in to me in the mornings to see how I am. They all come in just to say hello or if I'm in the breakfast room [of the hostel], they come there and say hello. It's just really nice, we're like one big family*. Sally, liver cirrhosis  *They did have a good manager down there [at the metal workshop hosted by a homelessness centre]. He had the patience of a saint, always find you something to do, so that was good because that kept me out of trouble and taking a lot more of the day, kept my drinking down.* Mark, liver cirrhosis  *I think they're taking me to Morrisons to have a look round and something to do. It stops me thinking about drink as well*. Sally, liver cirrhosis |
