# Supplementary material for: Normalization of alcohol misuse and alcohol-related harms: a mixed methods analysis exploring alcohol misuse, morbidity, and healthcare engagement in people experiencing homelessness
Source: Alcohol Alcohol. 2025 Dec 1;61(1):agaf071. doi: 10.1093/alcalc/agaf071 (PMC12667265; doi:10.1093/alcalc/agaf071)
Supplement: Supplementary_Topic_guide_1_agaf071 [file supplementary_topic_guide_1_agaf071.docx]

**SEMI-STRUCTURED INTERVIEW TOPIC GUIDE**

Introduction of the researcher, check the participant has read the information sheet and signed the consent form?

Does the participant have any further questions before the interview starts?

[Tape on]

Introductory questions

Tell me about yourself

Section 1: Health & alcohol

How is your health?

What do you think about your drinking (alcohol consumption)?

How do you think alcohol may be affecting your health?

How do those close to you influence your relationship with alcohol?

Section 2: Experience with Health care service utilisation and Hepatology outreach clinic

Describe how you use health care services?

What sort of things are you seen for?

How did you find the liver scan services that you had recently?

Can you explain why you agreed [or asked] to attend?

How does it compare to other experiences you have had with healthcare services?

How has having the scan affected you?

How has it made you think about your health?

Section 3: Experience with peer led referral coupons

What did you think when you were asked to bring a friend back with you to have a scan?

How did the offer of the £20 voucher affect what you thought?

What did you do after the offer was made?

If you did bring someone back:

1. Why did you do so?
2. What do you think that person thought when you asked them?
3. Why did they attend for the scan?
4. How has the experience changed your relationship with that person?
5. Why did you choose that person?

If you didn’t bring anyone back:

1. If you asked someone but they refused, do you know why?
2. If you didn’t ask someone, what stopped you from doing so?
3. If you didn’t ask someone is there anything that would have encouraged you to do so?

**Closing remarks**

Thank you for completing this interview. You have added to our understanding of the topics we have discussed. I don’t have any further questions but is there anything else you would like to ask?
